# Insurance barriers to substance use disorder treatment after passage of mental health and addiction parity laws and the affordable care act: A qualitative analysis

**DOI:** 10.1016/j.dadr.2022.100051

**Published:** 2022-03-31

**Authors:** Julia Dickson-Gomez, Margaret Weeks, Danielle Green, Sophie Boutouis, Carol Galletly, Erika Christenson

**Affiliations:** a Institute for Health and Equity, Medical College of Wisconsin, Milwaukee, WI, United States; b Institute for Community Research, Hartford, CT, United States; c Department of Psychology, University of Texas, Dallas, United States; d Center for AIDS Intervention Research, Medical College of Wisconsin, Milwaukee, WI, United States; e Center of Excellence in Women's Health, Boston Medical Center, BUSM, New England

**Keywords:** Parity laws, Substance use disorder, Medications to treat opioid use disorder, Methadone, Buprenorphine, Medicaid, ACA

## Abstract

**Introduction:**

People who use drugs (PWUDs) in the United States historically have had a higher probability of being uninsured. Passage of the Affordable Care Act, the Paul Wellstone and Pete Domenici Health Parity and Addiction Equity was expected to increase access to treatment for substance use disorder. Few studies to date have conducted qualitative research with substance use disorder (SUD) treatment providers regarding Medicaid and other insurance coverage of SUD treatment following passage of the ACA and parity laws. The present paper fills this gap by reporting data from in-depth interviews with treatment providers from three states, Connecticut, Kentucky, and Wisconsin, that differ in implementation of the ACA.

**Methods:**

Study teams in each state conducted in-depth, semi-structured interviews with key informants who provided SUD treatment, including providers of behavioral health residential or outpatient programs, office-based buprenorphine providers and opioid treatment programs [OTP, i.e. methadone clinics] (*n* = 24 in Connecticut, *n* = 63 in Kentucky and *n* = 63 in Wisconsin). Key informants were asked for their perceptions on how Medicaid and private insurance facilitates or limits access to drug treatment. All interviews were transcribed verbatim and analyzed for key themes using MAXQDA software using a collaborative approach.

**Results:**

Results from this study suggest that the promise of the ACA and parity laws to increase access to SUD treatment has only partially been realized. There is wide variation among the three states’ Medicaid programs and among private insurance in the types of SUD treatment that is covered. Neither Kentucky's nor Connecticut's Medicaid covered methadone. Wisconsin Medicaid did not cover residential or intensive outpatient treatment. Thus, none of the states studied here provided all levels of care that the ASAM recommends for treating SUD. Further, there were several quantitative limits placed on SUD treatment such as number of urine drug screens or visits allowed. Providers complained that many treatments required prior authorizations, including MOUD like buprenorphine

**Conclusions:**

More reform is needed to make SUD treatment accessible to all who need it. Such reforms should consider defining standards for opioid use disorder treatment with reference to evidence-based practices, not be attempting parity with an arbitrarily defined medical standard.

## Highlights

• The promise of the ACA and parity laws in increasing access to SUD treatment has not been realized.
• Medicaid does not cover all MOUD or SUD treatment levels in each of the three states.
• Medical necessity is used as a criterion to deny SUD treatment.
• Providers report significant administrative burden to billing medicaid and other insurance.

## 1 Introduction

People who use drugs (PWUDs) in the United States historically have had a higher probability of being uninsured, with approximately 26% of people with a substance use disorder (SUD) uninsured compared to 20% of the general public (Cummings et al., 2014; Garfield et al., 2010; Saloner et al., 2017). Further, before passage of the Mental Health Parity Act in 1996, the Paul Wellstone and Pete Domenici Health Parity and Addiction Equity Act in 2008, hereafter referred to as parity laws, and the Patient Protection and the Affordable Care Act in 2010, hereafter referred to as the ACA, many private insurance plans required higher cost sharing and special annual service caps limiting the number of visits or length of treatment available (Barry et al., 2016). The parity laws stipulated that if insurance plans covered behavioral health services, including treatment for SUD, they were required to cover them at a rate equivalent to comparable medical treatments. Additionally, many states passed their own parity laws (Barry et al., 2016). The ACA went further in requiring that all insurance plans included in the insurance exchanges and Medicaid include ten essential health benefits including treatment for substance use (Andrews et al., 2019).

It was expected that passage of the parity laws and the ACA would result in increased access to treatment for SUD both by increasing the numbers of PWUD who are insured, particularly with Medicaid expansion under the ACA, and by increasing coverage for SUD treatment. However, results of the ACA and parity laws on access to SUD treatment have been mixed (Abraham et al., 2017). Some states, particularly those that expanded eligibility to Medicaid to people making 138% of the federal poverty line, saw an increase in the number of PWUD who were insured (Knudsen et al., 2019). However, not all states elected to expand Medicaid. It has been estimated that 2.6 million uninsured adults with a behavioral health disorder failed to acquire coverage because they lived in a state rejecting Medicaid expansion, although the number of states opting to expand Medicaid has risen since the time of the study in 2018 (Rochefort, 2018). Data from a nationally-representative study of outpatient SUD treatment programs in 2013–2014, and 2016–2017 found that Medicaid expansion was associated with a 15.7 point increase in the percentage of patients insured by Medicaid in SUD treatment and a 13.7 point decrease in uninsured patients (Andrews et al., 2019). However, the overall number of patients receiving SUD treatment did not increase. States that covered intensive outpatient treatment and all medications to treat opioid use disorders (MOUD) reported a greater proportion of Medicaid patients in SUD treatment than states with more restrictive policies, such as requiring prior authorizations or quantity limits on services or medications (Andrews et al., 2019). Parity laws appeared to have more success in increasing access to mental health services than SUD treatment. The Substance Abuse and Mental Health Services Administration (SAMHSA) found that while spending by private insurance, Medicare, and Medicaid for mental health care increased between 1986 and 2014, spending for SUD treatment remained the same (Mark et al., 2016). SAMHSA also found that while the share of the population receiving mental health care increased after 2010, it remained stable for SUD treatment. Further, SUD treatment continues to be funded mostly by federal and state sources other than Medicaid (Garfield et al., 2010), while mental health care is most often financed by private insurance (Mark et al., 2014).

These mixed results suggest that the promise of the ACA and parity laws to increase access to SUD treatment has not been realized (Rochefort, 2018). Rochefort suggests that access to SUD treatment and mental health care did not increase, in part, because the aims of the reforms in the ACA and parity laws were actually quite modest (Rochefort, 2018). Parity laws, for example, don't mandate coverage for mental health or SUDs. Rather, they stipulate that *if* benefits for behavioral health services exist, they must be covered at the same level as equivalent medical benefits. In practice, equivalent coverage can be difficult to define because many behavioral health services and providers, such as residential (nonhospital) treatment, partial hospitalization, and peer support specialists, have no counterpart in general medical service (Garfield et al., 2010). In addition, parity is supposed to apply to both quantitative limits (e.g., number of visits), and non-quantitative limits (e.g., medical necessity of a procedure). However, some research has found that non-quantitative limits are imposed more frequently on mental health and SUD care than treatment for other health conditions (Legal Action Center, 2016).

Similarly, while the ACA requires coverage of SUD treatment, the level of private insurance in exchanges and Medicaid only needs to match coverage of equivalent medical conditions. Further, coverage for the “essential health benefits” which includes SUD treatment is determined based on “benchmark plans” that states can select, which then serve as a reference point (Berry et al., 2015; Corlette et al., 2015). Research has shown that some states offer much better behavioral health coverage under some benchmark plans than others. Less generous plans place limits on types of services, and type and severity of conditions (Mental Health America, 2015). Many plans have quantitative treatment gaps and formulary restrictions that appear to be out of line with parity laws. One study found that nearly one-quarter of plans offered on two state insurance exchanges appeared to violate federal parity laws regarding service authorization procedures and cost sharing (Berry et al., 2015). Jurisdiction for monitoring benefits of plans on the insurance exchange to make sure they meet ACA parity requirements and enforcement of noncompliance has not been specified in most states (Rochefort, 2018). Many plans require patients to visit specialty providers within their networks, further limiting access. A study that surveyed 3000 mental health and substance use consumers and their family members found more frequent denials for mental health and SUD treatment than for other medical care. In addition, 26% of those receiving insurance through health exchanges reported difficulties with locating mental health or substance use therapists or counselors that were in network, and 22% had difficulties finding a psychiatrist (National Alliance on Mental Illness, 2015). Many plans on insurance exchanges have very high deductibles leading to large out of pocket expenses (Allen et al., 2021; Manchikanti et al., 2017; Satre et al., 2020). Qualitative data collected from people seeking SUD treatment and SUD treatment providers can gain insight into the challenges of navigating insurance reimbursement.

Although Medicaid currently and historically has covered a wider array of SUD treatments than private insurance, individual states also determine what is covered under their Medicaid plans. For example, detoxification and methadone maintenance are not covered by some state Medicaid plans (Dahr, 2014; Mental Health America, 2015). Further, a longstanding prohibition against Medicaid payments to behavioral health facilities with more than 16 beds has restricted residential treatment in many facilities unless the state has received a waiver for this rule (Gorman, 2014). In a 2016 study, 21 states did not cover residential treatment and nine states did not cover intensive outpatient services (Grogan et al., 2016). This is in spite of the fact that the American Society for Addiction Medicine has determined, based on considerable scientific consensus and research, that four levels of care are required for effective SUD treatment: level 1 outpatient services, level 2 intensive outpatient services (IOP), level 3 residential inpatient services, and level 4 intensive inpatient services (American Society of Addiciton Medicine, 2021; Chuang et al., 2009).

Not surprisingly, given the still poor reimbursement for SUD treatment and regulatory barriers, some SUD treatment providers do not accept Medicaid or commercial insurance, particularly physicians who provide buprenorphine. In a national survey of buprenorphine-prescribing physicians, only 52% accepted Medicaid for buprenorphine-related office visits, with specialists in addictions and psychiatry less likely to accept Medicaid (Knudsen and Studts, 2018). Adequacy of perceived Medicaid reimbursement was positively associated with accepting Medicaid.

Few studies to date have conducted qualitative research with SUD treatment providers regarding Medicaid and other insurance coverage of SUD treatment following passage of the ACA and parity laws. Qualitative data can help illuminate people's experiences with reimbursement of SUD treatment including feelings of anger and stigma when claims are denied. The present paper fills this gap by reporting data from in-depth interviews with treatment providers from three states, Connecticut, Kentucky, and Wisconsin, that differ in implementation of the ACA. Connecticut and Kentucky both expanded Medicaid and established their own state insurance exchange. Kentucky had moved to the federal exchange in 2016 before the time of the study, 2018–2020. Wisconsin neither expanded Medicaid nor established its own marketplace. Perhaps as a result of this, Wisconsin relies much more on state and federal grants to fund SUD. Qualitative research can more readily explore the ways in which providers navigate insurance reimbursement for SUD treatment which may more accurately illuminate actual implementation of the ACA and parity laws. This can provide direct evidence of gaps in SUD treatment parity and suggest needed reforms.

## 2 Methods

### 2.1 Study overview

The current study is part of a larger project that aims to compare the factors that influence the effects of opioid-related laws and policies in Connecticut, Kentucky, and Wisconsin on the transitions from prescription opioids to heroin, fentanyl, and/or injection drug use. An urban, suburban, and rural area was selected in each state to examine the role of the local context on these transitions.

Study teams in each state conducted in-depth, semi-structured interviews with two groups: key informants and people who use heroin or prescription opioids nonmedically. The present paper uses data from key informants who provided SUD treatment, including providers of behavioral health residential or outpatient programs, office-based buprenorphine providers and opioid treatment programs [OTP, i.e., methadone clinics]. Interviews were conducted in 2018–2020. We identified an initial list of key informants using the expertise of the research teams located in each state. To be eligible, key informants had to be at least 18 years old and currently working in the specific sectors of interest which included, in addition to SUD treatment providers who are a focus of this paper, Medicaid regulators, first responders, PDMP (Prescription Drug Monitoring Program) regulators, drug court personnel and pharmacists. We used purposive sampling in each local area within each state to ensure that each of these roles was covered in the initial list. We then asked key informants for the names of additional people who occupied other key roles. We conducted 34 key informant interviews in Connecticut and 63 in both Kentucky and Wisconsin, for a total of 160 interviews. This paper uses data from 57 SUD treatment providers only. Of the SUD providers, 2 were in organizations that provided inpatient treatment, 16 residential treatment and 39 provided intensive outpatient. Approximately half of the providers used MOUD.

Potential participants were contacted by an email in which they were given a brief description of the study and told why they were being asked to participate. If these candidates expressed interest, interviewers scheduled a time to conduct a face-to-face interview when possible, or a phone interview. Only one interview was conducted by phone. All participants were told that their participation was voluntary and would be kept confidential and each provided written informed consent to participate in the study. Interviews were conducted by five researchers experienced in conducting in-depth interviews. Interviews lasted approximately 30 to 60 min and were audio recorded. All procedures were approved by the Institutional Review Board at the [Institution blinded for review]. Participants were not compensated for participation.

### 2.2 Interview content

Interview guides and probes differed depending on the sector to which participants belonged. All key informants were asked to describe their current job and responsibilities, and to assess the extent of prescription opioid, heroin and fentanyl misuse and factors that have contributed to it in their communities. The current paper focuses on key informants’ perceptions of how Medicaid and private insurance limited or facilitated access to drug tregatment and other ways of paying for SUD treatment.

### 2.3 Data analysis

All interviews were transcribed verbatim by professional, confidential transcription companies in each state. We used a collaborative approach for data analysis. First, we selected a transcript that was read by the multi-state research team to develop a preliminary list of codes. The preliminary coding list was then applied to three additional transcripts—which were purposively selected to reflect different experiences (e.g., the sector to which the key informant belonged, state, local area)— and refined until the research team reached consensus on a final list of codes, their meanings, and the procedures for assigning them to text data. The research team then used MAXQDA software to apply the final list of codes to the transcripts. The coding was completed by six members of the multi-state research team. Coding, development of new codes, and memoing (jottings done by coders to capture relationships between codes or initial hypotheses) were tracked by the six-person team. We also used bi-weekly team meetings for troubleshooting and quality checks that included the principal investigator of the study.

We used a constant comparative approach to analyze data for this paper. First, we identified quotes that focused on how insurance affected access to SUD treatment. We then compared how key informants from different states perceived insurance by focusing on SUD treatment providers from the different states. We also compared providers of buprenorphine, methadone, and behavioral health services, looking for variations in how insurance covered different forms of treatment, and the administrative burden of getting insurance reimbursement. Finally, we looked at other sources of funding for SUD treatment including federal and state block grants.

## 3 Results

Key informants interviewed included 32 women and 25 men. A little over half of the drug treatment programs in which participants worked accepted MOUD and around 44% of programs included residential treatment.

### 3.1 Benefit limits

#### 3.1.1 Type of treatment covered

Almost all providers reported that navigating insurance to pay for SUD treatment was a confusing and time-consuming process, and what kind of insurance a patient had in large part determined what kind of treatment he or she could receive. As one WI-based SUD treatment provider stated, “They tell us who we can treat, how we can treat them, they tell us how long we can see them.” Another SUD treatment provider stated:

So, we try to match her as much as we can, and to be perfectly honest, it's really based on where she can go with her insurance. (Wisconsin Behavioral Health provider, female)

Part of the reason that SUD treatment is matched to patients’ insurance rather than to their needs is because there is a wide variation in what insurance covers among plans within and between states. For example, while both Kentucky and Connecticut were Medicaid expansion states, neither state covered methadone treatment at the beginning of the study period. Wisconsin Medicaid, on the other hand, did not cover residential treatment, intensive outpatient, or, generally, medically supervised detoxification from opioids. Table 1 summarizes different treatments that were covered by the states at the time of this research.

**Table 1:** SUD Treatment Services Covered by Medicaid in Each State.

	Connecticut	Kentucky	Wisconsin
Inpatient detox	Yes	Yes	Yes for alcohol or benzodiazepines
Outpatient detox	Yes	No	No
Residential	Yes	Yes	No
Buprenorphine	Yes	Yes	Yes
Oral Naltrexone	Yes	Yes	Yes
Injectable Naltrexone	Yes	Yes	Yes
Methadone	No	No	Yes
Intensive Outpatient	Yes	Yes	No

Because methadone was not covered by Medicaid, providers in Connecticut and Kentucky stated that most of their clients paid out of pocket, which was a burden for most.

They don't have the financial resources to pay out of pocket; and their insurance doesn't cover it [methadone]. [The] price has gone up to almost $100 bucks a week. Now, if you think about it… it's $400/$500 a month. That's expensive. Yeah, I mean, people say, “Well, it's cheaper than heroin.” Yeah, but you don't have the same motivation. Do you want people breaking into your house to buy methadone or to get on the methadone program? (Connecticut OTP provider, male)

An unknown number may not have received services at all or elected to try buprenorphine which was covered by Medicaid. However, as mentioned by the provider below, methadone is a better option for some patients because of the added structure of daily, in-person dosing required.

Once word gets out that Medicaid's going to help pay for methadone treatment, we're going to have more people we'll help. They want help; they just can't pay for it… So, what do they do? They either continue using or they try something like Suboxone, which most of them have tried recovery with Suboxone. It's not for everyone. So, I foresee a bump of 50–100 patients easily. (Kentucky OTP, male)

It is not clear why Medicaid did not cover methadone treatment since it has decades of evidence of efficacy for treating OUD (Wakeman et al., 2020). Both Kentucky and Connecticut were moving toward Medicaid covering methadone during the study period.

#### 3.1.2 Medical necessity

Some treatments are not covered because they are considered to be outside of “medical necessity.” Wisconsin, for example, considers opioid withdrawal not to be life threatening, and it is therefore not covered under Medicaid.

[T]he reason why a lot of people can't stop using is when you stop using you go through withdrawal… And it's awful, and painful, and uncomfortable. And you go present at a hospital, and a hospital is going to say, "Well we're not going to admit you.” If it's opiate withdrawal, it's not medically necessary to treat it because you will not die from it. You will die from alcohol withdrawal. You'll die from benzodiazepine withdrawal. (Wisconsin Behavioral Health provider, female)

Most providers interviewed stated that treating withdrawal was not medically necessary, except in the case of pregnant women who may endanger the fetus if in withdrawal. Opioid withdrawal does not cause delirium tremens or other life-threatening conditions. However, as other providers pointed out, uncontrolled vomiting and diarrhea from withdrawal can be life threatening if it leads to dehydration. Further, withdrawal can increase the likelihood of relapse and overdose as PWUD go back to using illicit opioids to decrease withdrawal symptoms (Mark et al., 2003; Wines et al., 2007). In addition, detoxification is generally not warranted for MOUD that are opioid agonists like methadone and buprenorphine, although it is needed to initiate naltrexone, a medication used to treat opioid and alcohol addiction. This contraindication for medically supervised withdrawal may be another reason detox is not covered by Medicaid.

#### 3.1.3 Lack of equivalence of behavioral health services and medical care

Part of the difficulty in establishing parity between physical health and behavioral health services is that some behavioral health services have no obvious counterpart in general medical services. This can lead to seemingly arbitrary decisions about the rate at which services such as “peer support” will be covered. For example, as the woman below explains, reimbursement for peer support and group therapy was changed by Medicaid from being charged at an hourly rate to charging each session as “an event,” leading to great reductions in reimbursement rate.

Group therapy became an event… So, [before this change] if you had a group for an hour and a half, you could bill for an hour and a half. But like an event means if you see them for 15 min versus an hour and a half, you bill the same. So that hurt some of the programs.... The reimbursement rate went down to something like – don't quote me on this – to like $6.50 per person. So, if you have an hour group, and you get reimbursed $6.50 per person, and they put a limit, and you can only have eight people in a group. (Kentucky Behavioral Health provider, female)

MOUDs have an obvious counterpart to medications to treat other diseases, but behavioral services such as counseling do not, and are often seen as less “medically necessary” after the physical symptoms of withdrawal or cravings are controlled by medication.

But the end issue is, is they cut off service because they base most of their criteria off of a scale on the strictly medical-physical aspects of the drug use…. So, if they have no withdrawal symptoms or complications, their service levels will get cut very fast. (Wisconsin Behavioral Health provider, male)

#### 3.1.4 Reimbursement rates and limits on screenings

Further complicating things, Medicaid is administered by Managed Care Organizations (MCOs). Some people, generally those who were eligible for Medicaid before expansion, may receive Medicaid without MCOs. Different Medicaid MCOs within the same state can have different reimbursement rates for the same services.

Just the whole Medicaid issue in and of itself for payment. That has been a nightmare for us. Believe it or not, it just about sunk us a couple times…. If it was straight Medicaid, because each MCO is different. Like I said, they only pay for a limited number of screens, which I've written down here. And then, I don't know – they pay different amounts. One of the MCOs will pay $45.00 for a drug screen if you run it on an analyzer. Another will pay $65.00. Why not just have it across the board? Because we have to have budgets. (Kentucky Behavioral Health provider, female)

Many participants said that billing Medicaid was a full-time job and that claims were routinely denied and needed to be appealed, creating significant administrative burdens and hardship for SUD treatment programs.

In addition to covering certain kinds of treatment and not others, Medicaid and other insurance can set limits on the number of treatment sessions or screenings that are allowed. As described by the provider below, sometimes the number of urine drug screenings is limited by insurance, which can impede providers’ monitoring adherence to MOUD or from meeting the requirements of drug courts.

It's very hard to find providers in this field because of the stigma and then the providers feel like they're scrutinized. So, they want to test clients very frequently and the Medicaid does not want to reimburse, maybe once a month; so, 13 drug screens, usually, a year. But that really puts constraints on us to have a financial loss because we have to test the clients in order to have a great standard of care for the clients, and make sure there's no diversion and those sort of things. [KY Behavioral Health provider, female)

Some providers described getting prior authorization for particular treatment lengths and having to ask for more when that initial amount has been completed, as described below.

But what quite often happens, though, is we get an average about 10 days of PHP [partial hospitalization], right? 12 days. You know, that's the average…. You get lucky sometimes where you're able to push, push, push, and then they'll say, “No, we'll give you only three more days,” right? They'll come back to us. And the review process for this – the extensiveness of how much paperwork a therapist has to do versus the amount of time that they actually get to spend with the patient, it's a 50–50; 50 percent paperwork, 50 percent patient if you're in an environment like ours because you have to battle them for that time. (Wisconsin Behavioral Health provider, male)

Providers also complained that claims were often denied and that providers had to go through lengthy appeals processes to get paid. They argued that this was true not only of Medicaid but private insurance as well.

Interviewer: Other barriers that you've faced in terms of dealing with insurance companies? And you talked about kind of denying treatments or what other types of – Participant: Just that alone is every day. Every single patient. It's not one or two. It's every single patient cannot get enough care for one reason or another. The insurance company, if they don't deny you on the front end, they will kick you back on the back end and they will automatically kick it back and make up some reason…. Them saying that you don't have prior authorization when you did. Now, you gotta go back and fight them…. They try very hard to not pay you. (Wisconsin Behavioral Health provider, male)

This sense of always having to fight to get paid can help fuel burnout and feelings of being stigmatized for treating a stigmatized disease.

#### 3.1.5 Finding addiction specialists

Having MCOs manage Medicaid and private insurance in the exchange also means that providers must be within the MCO network to get reimbursed. In addition, some insurance requires those who provide SUD treatment, such as physicians who provide office-based buprenorphine, to specialize in psychiatry or addiction medicine.

In rural areas, they're now trying to say as far as for substance abuse, doctors need to be addictionologists or psychiatrists…. When you're in Louisville, Cincinnati, that's all and good. You've got those physicians pretty much everywhere…. When you're in rural Kentucky, you're very limited on a psychiatrist and an addictionologist. (Kentucky Behavioral Health provider, female)

### 3.2 Providers’ acceptance of insurance

Not surprisingly given the administrative burden in billing Medicaid and private insurance for treatment services and the low reimbursement rates, some providers did not accept insurance. This was especially true for providers who had their own small practices, often psychiatrists or primary care physicians.

Interviewer: Do you bill insurance through your practice?

Participant: I do not. I have tried over the years to do that, and I've failed. I think a solo practice would have to have like a full-time billing person to do that. And you have to fight for the money…. And it just doesn't work out in a solo practice. (Kentucky, buprenorphine provider, male)

Some healthcare systems would only accept patients from their own practices. As the participant below explained, this was because their SUD treatment services generally operated at a loss.

Our biggest requirement is that they have to be connected to our organization. So, historically, we treated the community at large, we just can't afford to anymore because of the poor reimbursement, and the demand for our services. We have a pipeline of referrals coming from our own organization. (Wisconsin, Behavioral health provider, male)

Many of the larger health systems did not cover SUD treatment for any but their own patients, leaving SUD treatment for those on Medicaid or without insurance to non-profits.

[Insurance is] the biggest reason why a lot of larger health systems don't provide significant outpatient behavioral health services. It's the reason [Local Hospital] won't even sit a toe in that water because they're a for-profit entity…. They're investing in residential inpatient partial hospitalization programs services. Cranking out a bunch of patients that need outpatient services, but not providing them themselves because there's no money in that. (Wisconsin Behavioral Health provider, male)

Insurance barriers get in the way of appropriate medical care in these instances, because for profit hospitals don't pay for all types of treatment or for follow-up care from inpatient residential treatment. Rather, patients are referred out of system for outpatient or intensive outpatient care where they may or may not be able to gain access due to limitations in number of treatment spots available. Follow-up treatment is generally recommended for patients who finish residential treatment services (American Society of Addiciton Medicine, 2021; Center for Substance Abuse Treatment, 2006).

### 3.3 Grant funding

In Wisconsin, and to a lesser extent Connecticut and Kentucky, federal block grants and non-Medicaid state funding cover many of the gaps in the coverage provided by Medicaid or other insurance. In Wisconsin, this means covering the kinds of treatment that Medicaid will not such as residential treatment. However, there are still limits on how long a person can stay in treatment. With block grants, rather than medical necessity, the state determines eligibility for a residential level of care based on “imminent danger,” meaning that the patient is a danger to him/herself or others. This is considerably stricter than medical necessity, but in practice can be difficult to determine because of the extreme social deprivation including homelessness that patients face as described by the provider below.

In that ASAM that I talk about, they talk about imminent danger and if you're in imminent danger you need to be in the residential level care. Once imminent danger is lessened, you can move to a lower level of care. How imminent danger is defined and looked at is much different for us than how they look at it… We're worried about not only her mental health, [but also] her ability to parent, the ability for her children to be safe, and all these other variables. I think Milwaukee County is very strict in how they look at imminent danger, and the ASAM kind of lays it out that way too. But it really wasn't laid out for somebody who's in poverty who has nothing. It's not like somebody who is middle class or upper that they go to 30 days of treatment and then they can go home someplace, where they're totally supported and it's nice and it's not – they're not back in the neighborhood. (Wisconsin Behavioral Health provider, female)

Block grants are administered through counties in Wisconsin. Many counties are reluctant to take patients from other counties or pay for patients who live in their counties to attend treatment in other counties. This leads to an inefficient use of resources as some facilities are not run at capacity while others have wait lists.

We did a survey…on residential bed availability. And there's a lot more beds available than are being filled. And part of that is because of the way our system is set up. It's very county oriented. We have open beds in this state and we really need a centralized location for not only assigning or getting people into available beds, but state-wide funding system. So, that the person up in Price County who needs residential level of care has just as much luck at getting it as somebody who lives in Milwaukee County. … Milwaukee County will not contract with these beds available in Washington County, let's say, but yet there these beds sit. So, that's a problem. (Wisconsin Behavioral Health provider, female)

Particular conditions or populations, such as those with OUD or who are pregnant, are prioritized under federal block funding in Wisconsin. In practice, this means that not everyone who needs treatment will get it.

Obviously, we can't pay for the world to go through treatment. So, if you're in another county, chances are you might not be funded for us unless you're pregnant. Unless you're at, maybe, a Chapter 51. You know what that is, that's a mandated you must go to treatment kind of situation. You're either a harm to yourself or harm to others. (Wisconsin Behavioral Health provider, female)

Given the strict criteria for using county funding, and its fragmentation across counties and priority populations, federal block grants and non-Medicaid state sources of funding are not likely to be a viable alternative to Medicaid and other insurance coverage.

## 4 Discussion

Results from this study suggest that the promise of the ACA and parity laws to increase access to SUD treatment has only partially been realized (Rochefort, 2018). There is wide variation among the three states’ Medicaid programs and among private insurance plans in the types of SUD treatment that is covered (Grogan et al., 2016). At the time these interviews took place, neither Kentucky's nor Connecticut's Medicaid covered methadone. Wisconsin Medicaid did not cover residential or intensive outpatient treatment, or to a large extent, detoxification from opioids. Thus, none of the states studied here provided all levels of care that the ASAM recommends for treating SUD. Further, there were several quantitative limits placed on SUD treatment such as number of urine drug screens or visits allowed (American Society of Addiciton Medicine, 2021; Chuang et al., 2009). Providers complained that many treatment modalities required prior authorizations, including MOUD like buprenorphine, and claims were frequently denied only to be accepted after appeals.

Many of the same kinds of barriers that existed to SUD treatment before ACA and parity laws remain such as limits on the type of SUD treatment provided, and the length of treatment. Further, results from our study illustrate the providers’ frustrations with navigating funding for SUD treatment with Medicaid and other insurance plans, a result of the still fragmented nature of SUD treatment and funding. The administrative burden in getting reimbursed for SUD treatment services may add to the stigmatization of SUD treatment and add to the burn-out and turn-over of staff in SUD treatment facilities. This, in turn, can further limit treatment access. SUD behavioral health treatment centers may be short staffed as a result of turn-over, limiting the number of patients that can be seen. Office-based buprenorphine providers may decide that they no longer want to prescribe buprenorphine to patients.

Parity of SUD treatment with other medical treatment has been made difficult by the fact that coverage for SUD and mental health, if offered, must only be equivalent to coverage for comparable medical treatment (Garfield et al., 2010). The problem is that many behavioral health services, such as residential treatment, intensive outpatient therapy, or peer support specialists, have no obvious counterparts in medical treatments. This is possibly why reimbursement for peer support specialists changed from an hourly to a flat rate as a Kentucky provider complained. Further, level of coverage is often calculated based on supposed “medical necessity” which appears to be somewhat arbitrarily defined and punitive to people with SUD (Legal Action Center, 2016). While diarrhea and vomiting from opioid withdrawal may not in all cases be life threatening, it is not clear whether insurance plans would deny coverage of treatment for the same symptoms that were caused by viral infection based on “medical necessity.” More problematic is denial of coverage for intensive outpatient and residential treatment. Results from this study suggest that the SUD treatment guidelines should be changed from “parity” with coverage for other medical conditions to a minimum level of coverage for SUD treatment that is in line with best practice. This should include, at a minimum, all levels of treatment recommended by ASAM and sufficient urinalysis to comply with court-ordered treatment and to monitor for buprenorphine metabolites to ensure that buprenorphine is being taken as prescribed and not diverted.

Given that MOUD has the highest evidence for efficacy for treating OUD and that methadone is the oldest MOUD available with the most evidence of efficacy (SAMHSA, 2014), it is troubling that Medicaid in Connecticut or Kentucky did not cover methadone. Medicaid in both states covered buprenorphine; thus, maybe offering one type of MOUD was considered sufficient. However, many patients who do not do well on buprenorphine do better on methadone and vice versa (Carroll et al., 2018), indicating that covering both kinds of treatment is best practice. Both Kentucky and Connecticut had plans to cover methadone during the study period. However, as of this writing Kentucky's Medicaid still did not cover methadone. The lack of coverage caused financial burdens for patients on methadone in both states and may have led an unknown number not to begin methadone treatment in the first place.

Managed care organizations (MCOs) have become a feature of Medicaid and private insurance (Bachman et al., 2004). MCOs work to manage care to limit costs, while theoretically keeping the quality of healthcare high. In practice MCOs often require patients to seek care within network and, in some cases, to see psychiatrists and physicians with specialties in addiction medicine to access office-based buprenorphine treatment. This can reduce access to treatment as there is a shortage of providers in addiction medicine or psychiatry, especially in rural areas. Indeed, previous research has shown that patients and families of patients with SUD often have a hard time finding providers within network (National Alliance on Mental Illness, 2015). Telemedicine use increased dramatically during the COVID-19 pandemic, particularly for behavioral health services. Expanded use of telemedicine required policy changes such as relaxing HIPAA requirements to allow telemedicine, insurance coverage at equal rates with in-person consultation, allowing buprenorphine induction by telemedicine consultations and expanding methadone take-home doses (Andraka-Christou et al., 2021; Kedia et al., 2021; Lin et al., In press; Rockwell and Gilroy, 2020; Sugarman et al., 2021). Early research has indicated that telemedicine was acceptable to both providers and patients, although poor internet access and low computer literacy were barriers to telemedicine for some patients (Lin et al., In press; Sugarman et al., 2021). Future research is needed to determine the comparative efficacy of in-person versus telemedicine consultations and group and individual therapy sessions as well as the extent to which telemedicine practices will be continued post-pandemic to increase access in rural and underserved areas (Lin et al., In press).

Similar to other research, not all providers accept Medicaid or other health insurance (Knudsen and Studts, 2018), and many healthcare systems will only treat patients who have already been seen within their system. Decreasing the administrative burden of Medicaid and other plans while increasing reimbursement rates will likely increase the willingness of providers to accept Medicaid and other insurance, thus, increasing access to SUD treatment. Providers from large healthcare systems reported that they provided SUD treatment at a loss. Healthcare systems may provide SUD treatment for existing patients as it may reduce medical costs associated with untreated SUD in the long run; however, there is not the same benefit in treating patients outside their system.

Federal block grants and non-Medicaid state funding covered much of SUD treatment in Wisconsin due to Medicaid not covering particular treatments and fewer people getting Medicaid coverage as Wisconsin was not a Medicaid expansion state. State and county funding, for example, paid for residential and intensive outpatient treatment while Medicaid covered neither. However, grant funding is not a feasible alternative to Medicaid expansion and improved private insurance coverage for SUD treatment because there are several barriers that prevent all who need treatment from getting it. First, in Wisconsin, funding for SUD was at the county level and access to SUD treatment in counties other than the one in which a patient resides was limited. Second, eligibility for residential treatment was limited to those who were in “imminent danger” of harming themselves or others which is even more restrictive than “medical necessity.” Finally, grant funding prioritizes certain populations and disordered use of particular substances to the exclusion of others. For example, pregnant women using opioids were a prioritized population due to the danger of opioid withdrawal to the fetus. This could limit access to others who may need it.

While the current study has a number of strengths including using qualitative methods to explore SUD treatment providers’ experiences with Medicaid and private insurance reimbursement and the use of a large sample of SUD treatment providers from three states including those who provide SUD treatment and those who do not, the study is not without limitations. The data from this study were collected from three states and may not be generalizable to other states Medicaid programs or insurance marketplaces. Further, in-depth interviews were conducted primarily to explore how differences in states’ laws and policies influenced transitions from prescription opioid use to heroin, fentanyl and injection drug use, and how differences in the availability and accessibility of SUD treatment and harm reduction may have affected drug use trajectories. Questions about experiences with Medicaid and other insurance providers were focused on the general topic of access to SUD treatment, not on changes to access since implementation of the ACA and parity laws. Thus, it is difficult to determine which issues were present before ACA and parity law implementation. However, participants’ discussion of “medical necessity” appears to refer to guidelines that were set by the parity laws and ACA as this was the standard used to determine coverage. Finally, data were collected in 2019–2020 and the situation regarding Medicaid and private insurance on insurance exchanges may have changed.

## 5 Conclusions

More reform is needed to make SUD treatment accessible to all who need it. Such reforms should define standards for OUD with reference to evidence-based practice for SUD, not by attempting parity with an arbitrarily defined medical standard. Continued research is needed to document SUD providers’ experiences with Medicaid and other insurance plans. Qualitative research is particularly well suited to exploring the implementation of ACA and parity laws to further improve access to SUD treatment.

## Author disclosures

**Role of funding source:** The opinions stated in the article are the authors’ and NIDA had no role in the preparation of this manuscript.

**Contributors:** All authors were involved in data analysis and read and approved the manuscript.

## Conflict of Interest

Nothing to declare.
